# Carotid artery plaque screening using abdominal aortic calcification on lumbar radiographs

**DOI:** 10.1371/journal.pone.0209175

**Published:** 2019-01-07

**Authors:** Kazuyoshi Kobayashi, Kei Ando, Taisuke Seki, Takashi Hamada, Koji Suzuki, Naoki Ishiguro, Yukiharu Hasegawa, Shiro Imagama

**Affiliations:** 1 Department of Orthopaedic Surgery, Nagoya University Graduate School of Medicine, Aichi, Japan; 2 Faculty of Medical Technology, School of Health Science, Fujita Health University, Aichi, Japan; 3 Department of Rehabilitation, Kansai University of Welfare Science, Osaka, Japan; Beijing Key Laboratory of Diabetes Prevention and Research, CHINA

## Abstract

**Aim:**

Arteriosclerotic disease is increasing due to aging of the population, and is associated with diabetes, hypertension, hyperlipidemia, obesity, and smoking. This disease may result in fatal cerebrovascular disease, and especially cardiogenic cerebral embolism caused by artery plaque-based atherothrombotic cerebral infarction. The study was performed to examine the relationship of abdominal aortic calcification (AAC) on lumbar radiographs with carotid intima-media complex thickness (IMT), factors associated with carotid artery plaque, and cutoff values in middle-aged and elderly people.

**Patients and methods:**

The subjects were 309 healthy volunteers (average age 63 years) who attended a health checkup supported by a local government in 2015. The AAC-24 score was determined on lumbar lateral standing radiographs and was categorized as 0 (54% of subjects),1–4 (31%), and ≥5 (severe, 15%). Carotid ultrasonography was used to evaluate IMT of the common carotid artery. Carotid artery plaque was defined as IMT >1.1 mm. Body mass index (BMI), hypertension, diabetes mellitus (DM), dyslipidemia, smoking, alcohol intake, and osteoporosis were examined.

**Results:**

Of 309 cases, 142 (46%) had AAC and 104 (34%) had carotid artery plaque. Thus, 15% (n = 45) had severe AAC. Age, prevalence of DM and carotid artery plaque increased with severity of AAC. In patients with carotid artery plaque (n = 104), age (67.8±7.5 vs. 61.0±10.1 years), % male (56% vs. 39%), BMI (22.9±2.8 vs. 23.7±3.5), AAC rate (58% vs. 40%) and AAC-24 score (3 (0, 8) vs. 0 (0, 2)) were all significantly higher than in those (n = 205) without carotid artery plaque. In multivariate analysis, age (OR 1.172), male gender (OR 1.654), AAC (OR 1.352), and AAC-24 ≥5 (OR 4.191) were significantly associated with carotid artery plaque. Combining AAC-24 with age significantly increased the AUC from 0.632 to 0.834 (p<0.05).

**Conclusion:**

There was a significant relationship between AAC on lumbar radiographs and carotid IMT.

## Introduction

Rapid aging of the population has increased the number of cases of arteriosclerotic disease [1]. This is a concern because this disease can lead to fatal cerebrovascular disease, with cardiogenic cerebral embolism caused by artery plaque-based atherothrombotic cerebral infarction and proliferation of non-valvular atrial fibrillation being especially critical [2]. Detection of the presence and progression of carotid artery plaque is normally performed by carotid ultrasonography, which is used to measure intima-media complex thickness (IMT) as a marker of carotid artery plaque and a risk factor for cardiovascular events [3,4]. IMT that is equal to or thicker than an absolute threshold or a predicted IMT based on age and other covariates is considered to indicate atherosclerosis [5–8].

Arteriosclerotic lesions including coronary artery disease, aortic disease and cerebrovascular atherosclerotic disease are also fatal, and early diagnosis is desirable. Kauppila et al. reported lumbar radiography as a convenient method for evaluation of abdominal aortic calcification (AAC) [9], and an association between AAC and cardiac disease has been reported [10–12]. However, the relationship of AAC with carotid IMT has not been examined. If simple screening of carotid artery plaque is possible using lumbar radiographs, it may be helpful for prevention of cerebrovascular disorders. Furthermore, lumbar radiographs are recorded in patients scheduled to undergo spinal surgery, and prediction of carotid artery plaque from AAC may be useful for perioperative risk management in these patients. Therefore, the purpose of this study was to examine AAC found in lumbar radiographs, carotid IMT and factors associated with carotid artery plaque, and to detect an AAC cut-off value for prediction of carotid artery plaque in middle-aged and elderly people.

## Patients and methods

**Fig 1:**
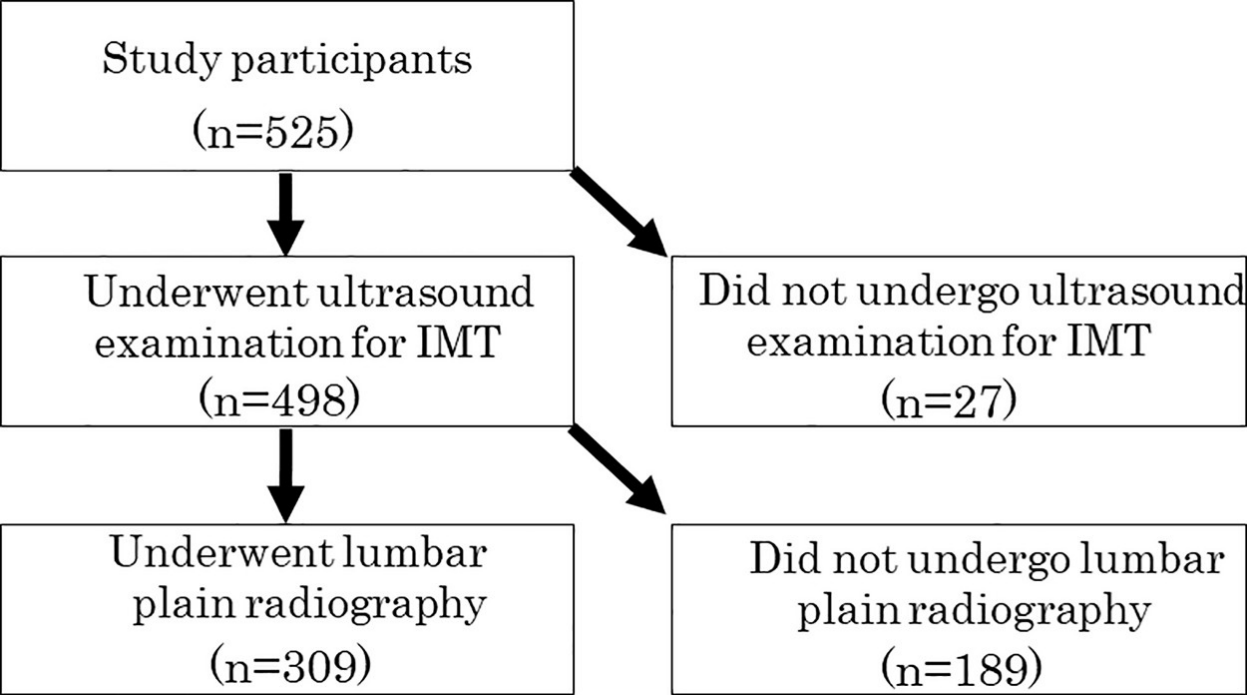
Selection of study participants.

The subjects were healthy volunteers who attended a basic health checkup supported by a local government in 2015. This checkup has been held annually in the town of Yakumo for 34 years and includes voluntary orthopedic, physical function, and internal medical examinations [13,14]. The inclusion criteria were Japanese men and women aged older than 40 years who underwent radiographs of the lumbar spine and consented to participate in the study. Of 525 individuals who underwent the checkup in 2015, the current study was performed in 309 (average age, 63.3 years; age range, 40–88 years; 137 men and 172 women). AAC was measured on lumbar lateral standing radiographs and IMT of the common carotid artery (CCA) was determined by ultrasound examination (Fig 1).

Body mass index (BMI), hypertension, diabetes mellitus (DM), dyslipidemia, smoking, alcohol intake, osteoporosis and carotid artery plaque were examined as follows. Trained nurses administered a questionnaire on health and daily lifestyle habits, including smoking (current smoker, ex-smoker, or nonsmoker), and alcohol habit (drinking at least once a week, or none) [15,16]. The responses for alcohol intake were used to divide the subjects into those with a history of alcohol intake and those who had never drank alcohol. Anthropometric indices (height and weight) and blood pressure were measured during the health examination. BMI was calculated as body weight (kg) divided by height (m) squared. Bone mineral density (BMD) was ultrasonically measured in the calcaneus using a bone densitometer (A1000 Insight, Lunar Corp., Madison, WI, USA), and the percent of the young adult mean (%YAM) was measured. Diagnosis of osteoporosis was based on the criteria of the Japanese Society for Bone and Mineral Research [17], and was defined as a percentage of the %YAM <70% in the calcaneus [13,18]. Hypertension was defined as systolic blood pressure >140 mmHg and/or diastolic blood pressure >90 mmHg, based on guidelines of the Japanese Society of Hypertension [19], or use of antihypertensive medications. DM was defined as current use of oral hypoglycemic agents, insulin, or a self-reported diagnosis. Dyslipidemia was defined as triglycerides ≥150 mg/dl, HDL-C <40 mg/dl, or LDL-C ≥140 mg/dl, based on the guidelines of the Japan Atherosclerosis Society [20], or use of antidyslipidemic drugs.

Relationships between AAC in lumbar radiographs and arteriosclerosis-related factors (age, sex, BMI, hypertension, DM, dyslipidemia, smoking (current and previous), alcohol habit, %YAM, osteoporosis and carotid artery plaque) were examined. All participants provided written informed consent, and the study was approved by the Committee on Ethics in Human Research of our University. The study procedures were carried out in accordance with the principles of the Declaration of Helsinki.

### Assessment of abdominal aortic calcification

**Fig 2:**
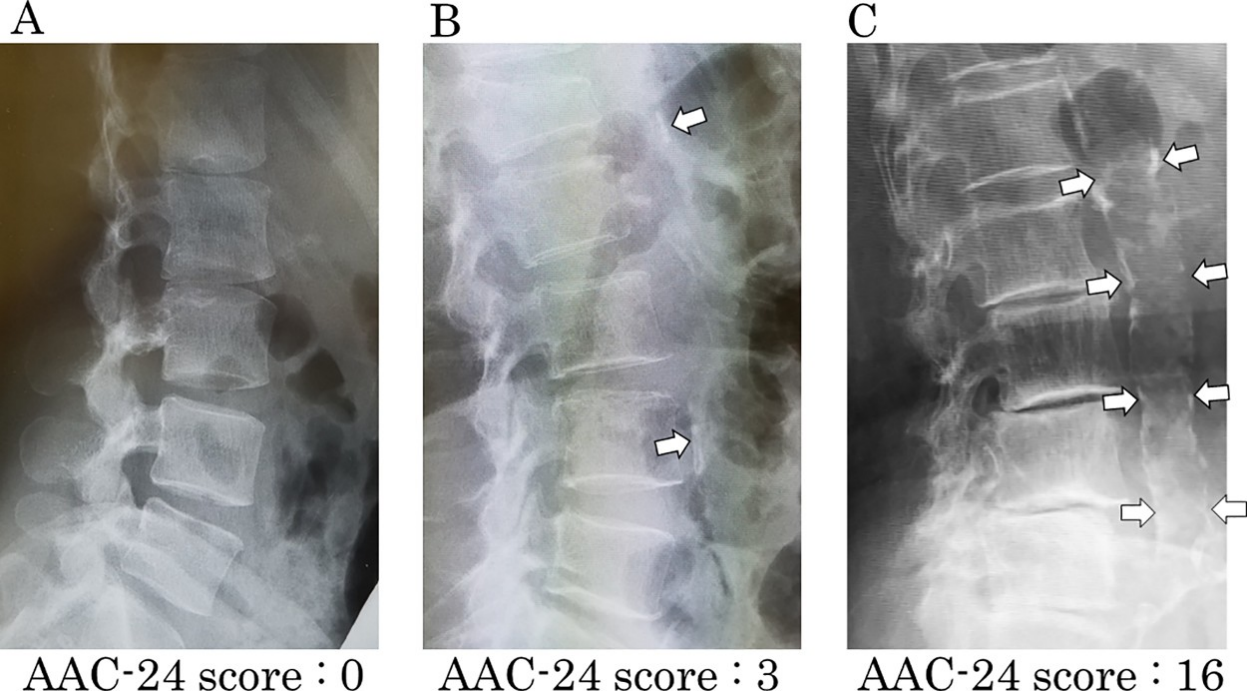
Radiographic images with (A) no abdominal aortic calcification (AAC), (B) moderate AAC (AAC-24 score = 4), and (C) moderate AAC (AAC-24 score = 16). AAC is indicated by white arrows.

Aortic calcification was assessed only on lateral films of the abdominal aorta and lumbar spine because of difficulties in assessing thoracic aortic calcification. A standard technique for lateral lumbar spine radiographs in a standing position was used, using a 100-cm film distance, 94 kVP, and 33–200 mAs [21,22]. The AAC-24 score was used to quantify calcification on lumbar radiographs (Fig 2), using a method discussed in detail in elsewhere [12,23]. Briefly, in the AAC-24 system, the anterior and posterior aortic walls are divided into four segments corresponding to the L1-L4 areas in front of the lumbar vertebrae, as described by Kauppila et al. [9] (Table 1). Aortic calcification is scored as 0 (no calcification), 1 (≤ 1/3 of the aortic wall in that segment calcified), 2 (>1/3 to ≤ 2/3 of the aortic wall calcified), and 3 (>2/3 of the aortic wall was calcified). Scores range from 0–6 for each vertebral level, and total score ranges from 0–24 [9]. Two authors (K.K. and S.I.) assessed AAC and determined the AAC-24 score by consensus.

**Table 1:** Classification of abdominal aortic calcification by Kauppila et al.[10].

Grade 0	No calcific deposits in front of the vertebra
Grade 1	Small, scattered calcific deposits filling less than one-third of the longitudinal wall of the aorta in front of the vertebra
Grade 2	One-third or more, but less than two-thirds of the longitudinal wall of the aorta calcified in front of the vertebra
Grade 3	Two-thirds or more of the longitudinal wall of the aorta calcified in front of the vertebra

### Carotid ultrasonography

**Fig 3:**
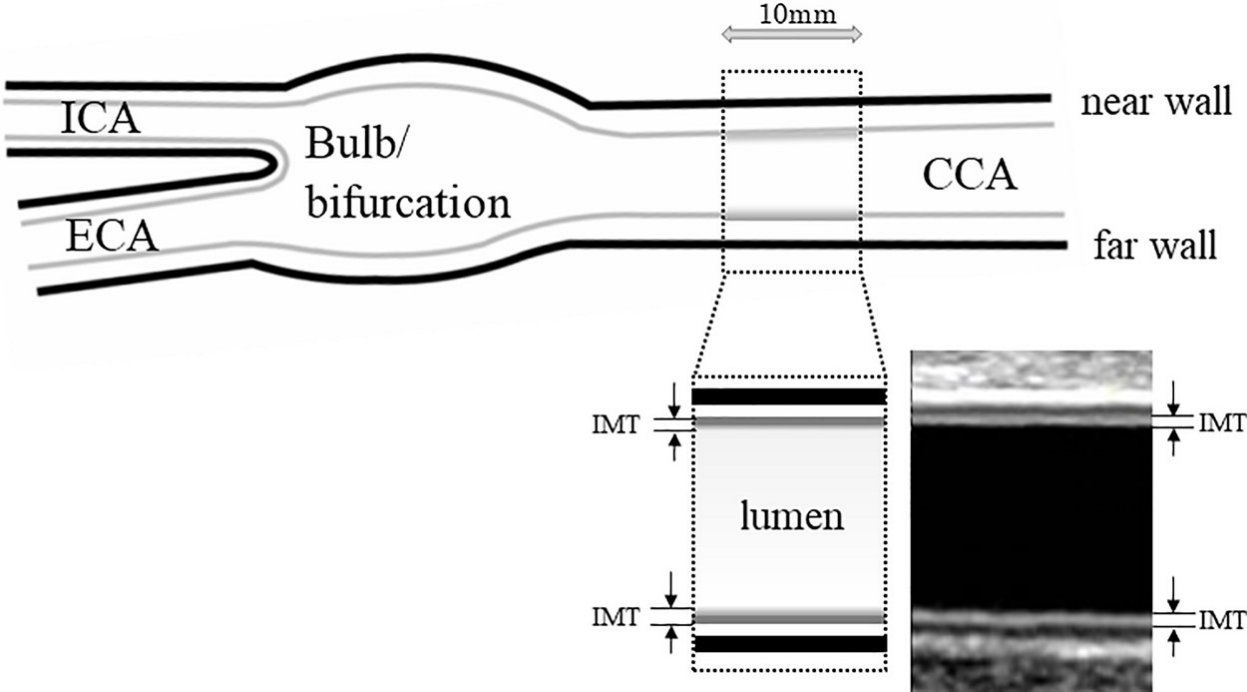
Measurement of intima-media thickness (IMT) in an ultrasonic image. CCA: common carotid artery, ICA: Internal carotid artery, ECA: External carotid artery.

The details of the examination methods have been described elsewhere [24]. An imaging study of the CCAs was performed using a high-resolution ultrasonic measurement system with a center frequency of 7.5 MHz. The user selects frames with good perpendicular alignment and image quality and adjusts the IMT box position if necessary to ensure measurement of the mean IMT over the distal 10 mm of the far wall of each CCA. For every participant, 5 to 10 mean IMT measurements were taken at the same phase of the cardiac cycle (diastole, electrocardiography gated) for each artery (right/left) [15] (Fig 3). IMT measurements from both arteries were averaged to give the final IMT. Carotid plaque was defined as IMT >1.1 mm^2^.

### Statistical analysis

Statistical analysis was conducted using SPSS ver.22 (SPSS Inc. Chicago, IL, USA). Non-normally distributed variables are presented as median (interquartile range (IQR)) and compared by paired Mann-Whitney test. Normally distributed variables are presented as mean ± standard deviation (SD) and compared by paired *t* test. Categorical variables were compared by Chi-squared test. Differences among three groups were analyzed by Bonferroni test and Kruskal-Wallis test. Univariate and multiple logistic regression was used to estimate the odds ratio and 95% confidence intervals (CIs) for potential predictors of carotid artery plaque, using AAC in lumbar radiographs and arteriosclerosis-related factors (age, sex, BMI, hypertension, DM, dyslipidemia, smoking, alcohol intake, %YAM, osteoporosis and carotid artery plaque) in patients with and without carotid artery plaque. Age and IMT were correlated with ACC-24 scores; therefore, Spearman correlation was used to control for the confounding effects of age and IMT. Net reclassification improvement (NRI) was derived from logistic regression models [25]. To assess the value of the age conventional and combined (age and AAC-24) probabilistic model further, we estimated the receiver operating characteristic (ROC) curves and area under the curve (AUC) or *c*-statistic with 95% confidence interval (CI) using corresponding logistic models. P < 0.05 was considered to be significant in all analyses.

## Results

**Fig 4:**
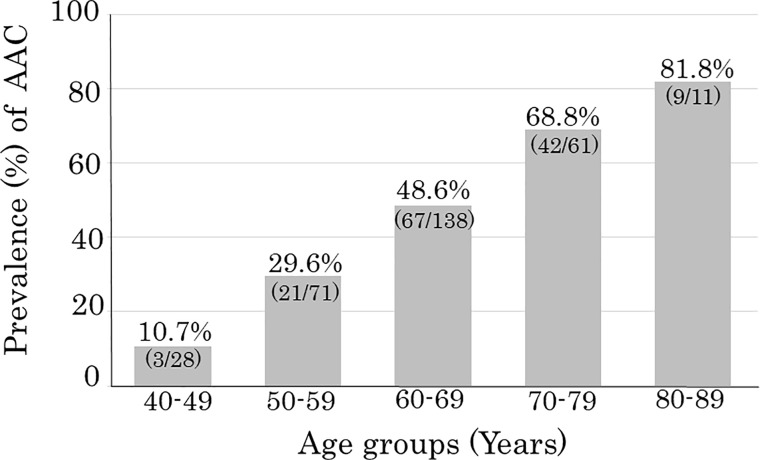
Percentage (%) of abdominal aortic calcification (AAC) by age group. The presence of AAC was assessed as shown in Table 1 (grades 1–3).

**Fig 5:**
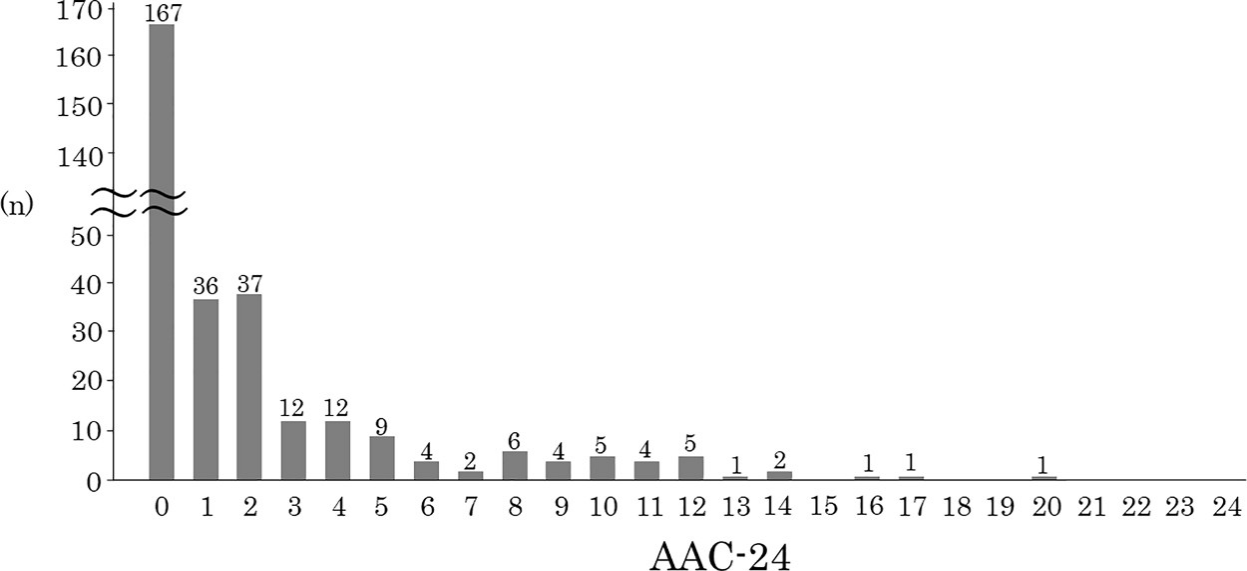
Distribution of AAC-24 scores in all participants (n = 309).

The mean values of measured variables in the 309 subjects (130 males and 179 females) are listed in Table 2. The average age was 63 years old. Of all 309 cases, 142 (46%) had aortic calcification and 104 (34%) had carotid artery plaque (Table 2). The prevalences of aortic calcification by age were 10.7% (3/28), 29.6% (21/71), 48.6% (67/138), 68.8% (42/61), and 81.8% (9/11) in subjects aged 40–49, 50–59, 60–69, 70–79, and ≥80 years old, respectively (Fig 4). AAC-24 scores were categorized into three groups of 0, (none) 1–4 (moderate), and ≥5 (severe), which corresponded to 54%, 31% and 15% of the subjects, respectively (Table 3). As severity of AAC increased, age and prevalence of DM and carotid artery plaque also increased. The distribution of AAC-24 for all participants is shown in Fig 5.

**Table 2:** Clinical background of the subjects (n = 309).

Item	Value
Age (years)	63±10
Male/female (n)	130/179
Body mass index (kg/m^2^)	23.6±3.4
Hypertension (n)	102 (33%)
Diabetes mellitus (n)	30 (10%)
Dyslipidemia (n)	96 (31%)
Smoking (n)	40 (13%)
Alcohol habit (n)	151 (49%)
% Young adult mean (%)	81±15
Osteoporosis (n)	70 (23%)
Abdominal aortic calcification (n)	142 (46%)
Carotid artery plaque (n)	104 (34%)

**Table 3:** Characteristics of the 309 cases by category of AAC-24 score.

Characteristic	AAC-24: 0 (54%, n = 167)	AAC-24: 1–4 (31%, n = 97)	AAC-24: ≥5 (15%, n = 45)	p
Age (years)	59.8±9.3	65.4±8.5	71.5±7.5*	<0.01
Male (n)	65 (39%)	45 (46%)	17 (38%)	n.s.
Body mass index (kg/m^2^)	23.6±3.5	23.5±3.1	23.3±3.2	n.s.
Hypertension (n)	49 (29%)	35 (36%)	18 (40%)	n.s.
Diabetes mellitus (n)	18 (11%)	4 (4%)	8 (17%)**	<0.05
Dyslipidemia (n)	57 (34%)	26 (27%)	13 (29%)	n.s.
Smoking (n)	18 (11%)	13 (13%)	7 (16%)	n.s.
Alcohol habit (n)	77 (46%)	55 (57%)	19 (42%)	n.s.
% Young adult mean (%)	83±14	79±15	77±13	n.s.
Osteoporosis (n)	36 (21%)	22 (23%)	12 (26%)	n.s.
Carotid artery plaque (n)	44 (26%)	33 (34%)	27 (60%)**	<0.01

AAC: abdominal aortic calcification

* Age was significantly greater for subjects with AAC-24 score ≥5 (p<0.01, by Bonferroni test)

** Diabetes mellitus and carotid artery plaque were significantly more frequent in subjects with AAC-24 score ≥5 (p<0.01, p<0.05 by Kruskal-Wallis test)

**Fig 6:**
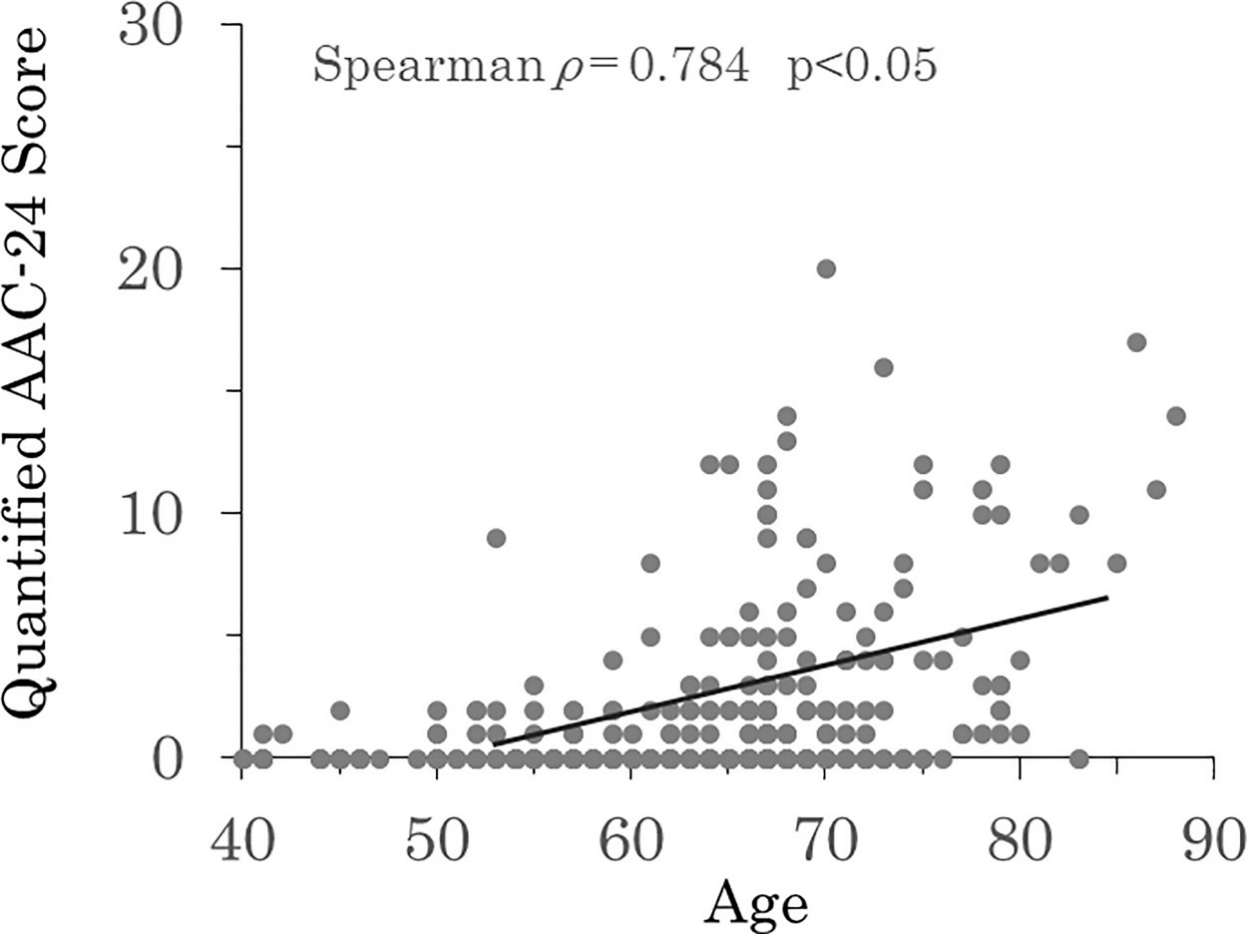
Relationship between AAC-24 scores and age (Spearman ρ = 0.784, p<0.05).

**Fig 7:**
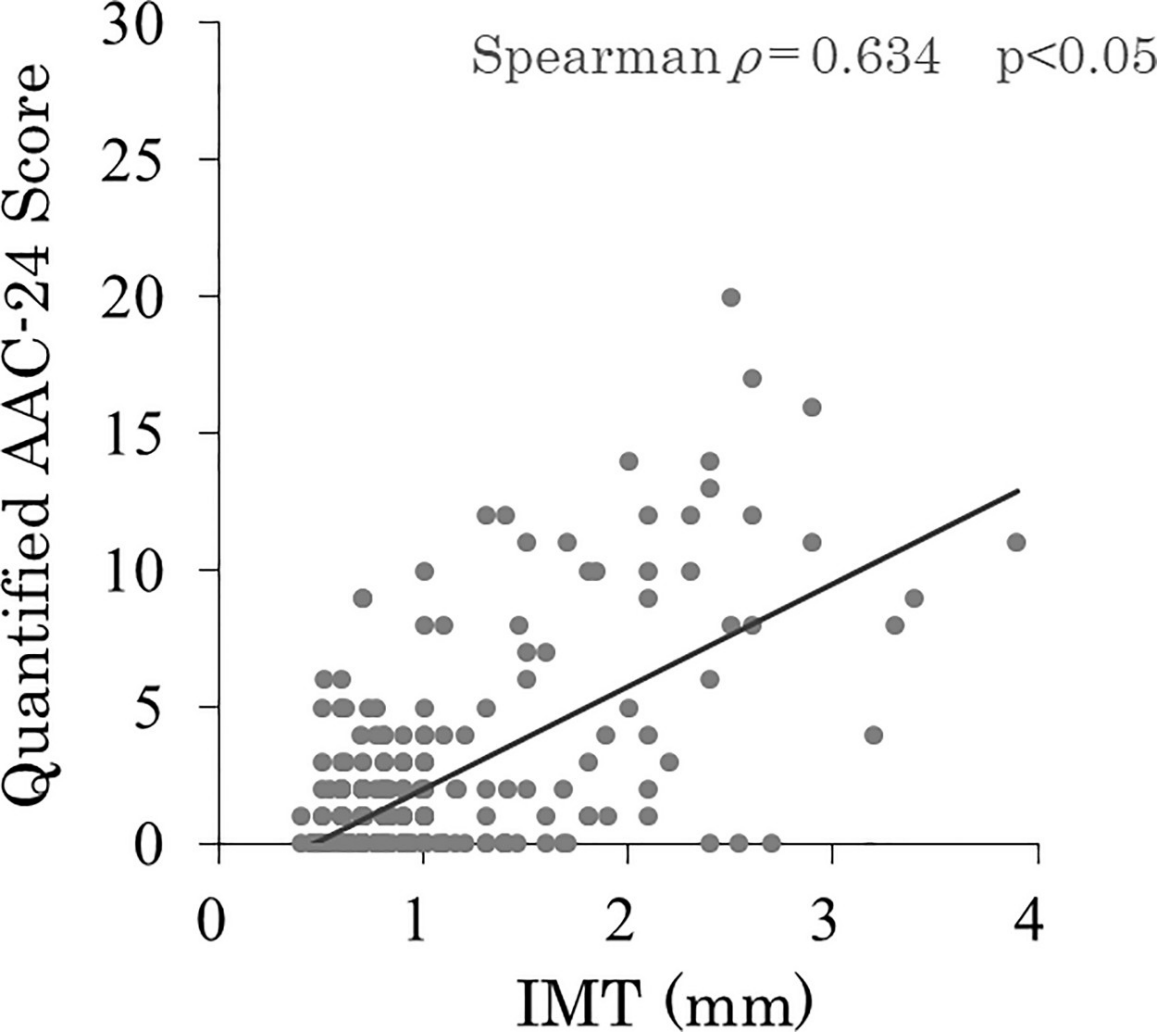
Relationship between AAC-24 scores and IMT (Spearman ρ = 0.634, p<0.05).

There were no significant differences in hypertension, DM, dyslipidemia, smoking, alcohol habit, %YAM and osteoporosis between patients with (n = 104) and without (n = 205) carotid artery plaque. Age (67.8±7.5 vs. 61.0±10.1 years, P<0.01), male gender (56% vs. 39%, P<0.01), BMI (23.7±3.5 vs. 22.9±2.8 kg/m^2^, P<0.05), AAC (58% vs. 40%, P < 0.01) and AAC-24 score (3 (0, 8) vs. 0 (0, 2), P<0.01) were significantly higher in patients with carotid artery plaque (Table 4). The results of univariate and multivariate analyses are shown in Table 5. In univariate logistic regression analysis, age (OR 1.274, 95% CI 1.065–1.325; P<0.05), male gender (OR 2.481, 95% CI 1.428–5.020; P<0.01), body mass index (OR 1.321, 95% CI 1.017–2.196; P<0.05), AAC (OR 1.525, 95% CI 1.244–2.748; P<0.01) and AAC-24 ≥5 (OR 5.933, 95%CI 2.304–9.475; p<0.01) were significantly associated with carotid artery plaque. In multivariate logistic regression analysis, age (OR 1.172, 95% CI 1.043–1.264; P<0.05), male gender (OR 1.654, 95% CI 1.021–2.694; P<0.05), AAC (OR 1.352, 95% CI 1.143–2.493; P<0.05), and AAC-24 ≥5 (OR 4.191, 95%CI 2.103–8.352; p<0.01) were significantly associated with carotid artery plaque (Table 5). AAC-24 scores had a significant relationship with age (Spearman *ρ* = 0.784, p<0.05; Fig 6) and IMT (Spearman *ρ* = 0.634, p<0.05; Fig 7).

**Table 4:** Difference in variables between subjects with and without carotid artery plaque.

Item	Carotid artery plaque (+) (n = 104)	Carotid artery plaque (-) (n = 205)	p
Age (years)	67.8±7.5	61.0±10.1	<0.01
Male (n)	58 (56%)	79 (39%)	<0.01
Body mass index (kg/m^2^)	23.7±3.5	22.9±2.8	<0.05
Hypertension (n)	38 (36%)	64 (31%)	n.s.
Diabetes mellitus (n)	8 (8%)	22 (11%)	n.s.
Dyslipidemia (n)	34 (33%)	62 (30%)	n.s.
Smoking (n)	17 (16%)	23 (11%)	n.s.
Alcohol habit (n)	62 (60%)	89 (43%)	n.s.
% Young adult mean (%)	79±17	81±14	n.s.
Osteoporosis (n)	30 (29%)	40 (20%)	n.s.
Abdominal aortic calcification (n)	60 (58%)	82 (40%)	<0.01
AAC-24 score (median (IQR))	3 (0, 8)	0 (0, 2)	<0.01

IQR: interquartile range

**Table 5:** Univariate and multivariate logistic regression analyses for carotid artery plaque.

	Univariate		Multivariate	
Variable	OR (95% CI)	p-value	OR (95% CI)	p-value
Demographics				
Age	1.274 (1.065–1.325)	<0.05*	1.172 (1.043–1.264)	<0.05*
Gender (male)	2.481 (1.428–5.020)	<0.01*	1.654 (1.021–2.694)	<0.05*
Body mass index	1.321 (1.017–2.196)	<0.05*	1.284 (0.984–1.935)	0.09
Abdominal aortic calcification	1.525 (1.244–2.748)	<0.01*	1.352 (1.143–2.493)	<0.05*
AAC-24 score (vs. 0)				
≥5	5.933 (2.304–9.475)	<0.01*	4.191 (2.103–8.352)	<0.01*

*p<0.05

OR, odds ratio; CI, confidence interval

**Fig 8:**
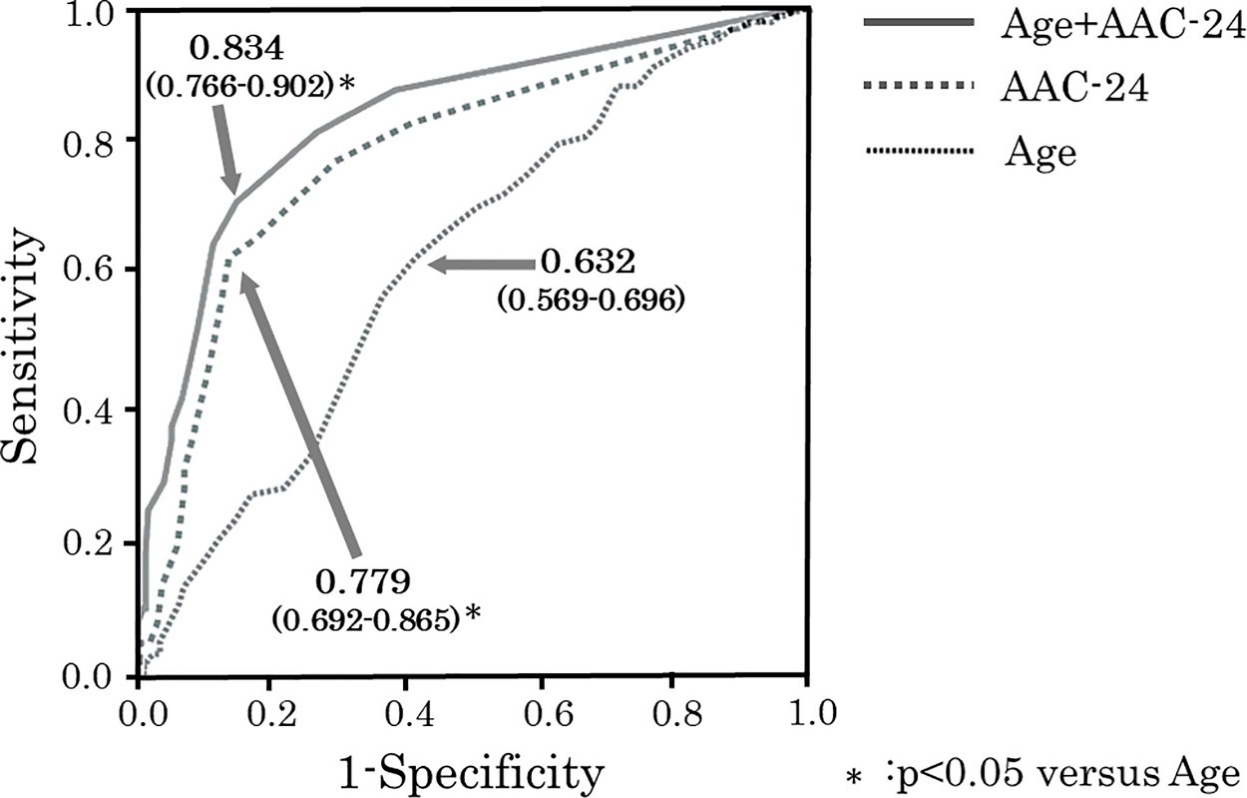
Receiver operating characteristic (ROC) curves for prediction of carotid artery plaque using AAC-24 scores, age, and a combination of these factors. Based on c-statistics, the area under the curve (AUC) for age was 0.632 [95% CI 0.569–0.696], and the AUC for AAC-24 was 0.779 [95% CI 0.692–0.865]. Combining AAC-24 with age significantly increased the AUC to 0.834 [95% CI 0.766–0.902].

In the total cohort (Table 6), reclassification based on models using age and AAC-24 in subjects with or without carotid artery plaque resulted in an NRI of 21.2% [95% CI 0.16–0.34; p<0.05]. ROC curves are shown in Fig 8. Based on c-statistics, the AUC for age was 0.632 [95% CI 0.569–0.696] and the AUC for AAC-24 was 0.779 [95% CI 0.692–0.865]. In ROC analysis, carotid artery plaque was predicted by an age of 66 with sensitivity 61% and specificity 59%, and by an AAC-24 score of 3.5 with sensitivity 63% and specificity 86%. Combining AAC-24 with age significantly increased the AUC to 0.834 [95% CI 0.766–0.902] (p<0.05) (Table 7, Fig 8).

**Table 6:** Reclassification of the total cohort based on models using age and AAC-24 scores in subjects with or without carotid artery plaque.

	Reclassification using AAC-24 scores			
Reclassification based on age	0	1–4	≥5	Total number
With carotid artery plaque				
<60	21	10	3	34
60–70	25	19	7	51
>70	10	5	4	19
Total number	56	34	14	104
Without carotid artery plaque				
<60	34	21	9	64
60–70	53	31	16	100
>70	24	11	6	41
Total number	111	63	31	205
Net reclassification improvement	21.2% (p<0.05)			

**Table 7:** C-statistic and measures and model fit for the conventional risk factors of age and AAC-24.

Risk factors and items	C-statistic (95% CI)	*P* value
Age	0.632 (0.569–0.696)	Reference
AAC-24	0.779 (0.692–0.865)	<0.05 (vs. Age)
Age + AAC-24	0.834 (0.766–0.902)	<0.05 (vs. Age)

CI, confidence interval

## Discussion

There are several previous reports of AAC evaluation on lumbar radiographs [9,23,26,27]. With respect to arteriosclerosis, the incidence of AAC increases with aging and is considered to be a risk factor for cardiovascular disease [12,23,28,29]. In particular, calcification of the coronary arteries is a common complication and could be a risk for myocardial infarction and sudden death [12,26]. AAC assessment on lumbar radiographs using a semiquantitative score is rapid, inexpensive and safe. Thus, it is easily available in clinical practice and provides useful information on current cardiovascular status and further cardiovascular risk. However, the relationship between carotid artery plaque and AAC has not been examined previously.

Carotid IMT is a surrogate marker for carotid artery plaque and an established index of chronic changes in atherosclerosis [30–33]. The clinical significance of increased plaque includes increases of cerebral infarction [34] and of coronary artery disease in an arteriosclerosis-related study [35]. Measurement of carotid IMT using ultrasonography is performed worldwide because it is simple, reproducible, and noninvasive. In a previous report of the clinical significance of IMT for cerebrovascular events, the incidence of cardiovascular disease was found to increase with an IMT of the CCA >1.18 mm in people aged ≥65 years [36]. In the Rotterdam study, the average carotid IMT was 1.17 mm in patients with myocardial infarction, 1.22 mm in those with stroke, and 1.02 mm in healthy controls [37]. Furthermore, IMT ≥1.2 mm in the CCA has a poor prognosis in cardiovascular disease [37], and in a large-scale study of hyperlipidemia and hypertension, the change in IMT of the CCA was also useful for judging the therapeutic effect [38]. However, it has also been stated that IMT ≥1.1 mm rarely occurs in healthy elderly people [2], and Barnett et al. suggested that IMT ≤1.0 mm is normal and IMT ≥1.1 mm is abnormal thickening [39]. In general, IMT that is equal to or thicker than an absolute threshold or a predicted IMT based on age and other covariates is considered to be plaque [5–8]. Thus, in view of all these factors, plaque was defined as abnormal IMT ≥1.1 mm in our series.

In our series, ROC analysis gave a cutoff value for the AAC-24 score of 3.5 for prediction of carotid artery plaque on a lumbar radiograph, and in multivariate logistic regression, an AAC-24 score ≥5 was significantly more frequent in patients with carotid artery plaque based on IMT. Lumbar radiographs are also frequently performed in patients scheduled to undergo spinal surgery. In previous reports, fatal perioperative complications after spinal surgery have included ischemic heart disease at rates of 0.7–2.9%. For prevention of this complication, a case with calcification in the abdominal arterial wall of more than one-third on a lumbar image should undergo preoperative cardiac function evaluation by a cardiovascular specialist [40]. Our results suggest that evaluation of AAC on lumbar radiographs can predict the presence of carotid artery plaque, and this finding could trigger additional screening using ultrasonography of the CCA in patients scheduled for spinal surgery, leading to increased efficacy in assessment of the risk of cerebrovascular disease. This may be particularly important because fusion surgery using instrumentation is increasingly common and has greater surgical invasiveness, including a larger intraoperative bleeding volume, which may increase the risk of perioperative complications. Thus, further studies are necessary to establish the potential utility of AAC obtained from lumbar radiographs for prediction of carotid artery plaque in clinical practice.

Regarding the degree of AAC, as shown in Table 3, significant associations were found with age, diabetes, and carotid artery plaque, whereas hypertension and dyslipidemia as comorbidities showed no significant association. In particular, the frequency of dyslipidemia was relatively high in the AAC-24: 0 group. This may have been because there were many middle-aged and elderly people in our series, and patients using antidyslipidemic drugs and antihypertensive medicines were still defined as having hypertension or dyslipidemia, even if their treatment control was good.

There are several limitations in this study. First, AAC was detected only on lumbar radiographs, and not by accurate diagnosis by CT; however, CT is not commonly used in an examination of healthy volunteers. Second, we focused on Japanese elderly and middle-aged people, and the determined cut-off values cannot be applied to all races. Third, the ideal endpoint is cerebrovascular events, but using this endpoint in the general population requires a longitudinal study. Fourth, using the cut-off AAC-24 score of 3.5 from ROC analysis, the specificity was 86%, but the sensitivity was only 63%. Therefore, a screening method using only AAC is not necessarily highly sensitive; even in patients without AAC, 26% of cases will be judged to have carotid artery plaque, which suggests that evaluation using AAC alone may be limited. Use of ultrasonography might be a better screening modality. However, this is the first report of a relationship between AAC on lumbar radiographs and carotid IMT, and our series is beneficial in that it includes healthy middle-aged and elderly people.

In conclusion, we examined AAC on lumbar radiographs, carotid IMT and risk factors for plaque, and obtained a cut off value of 3.5 for the AAC-24 score for prediction of carotid artery plaque in middle-aged and elderly people. In multivariate logistic regression, AAC-24 scores ≥5 (OR 4.191) were significantly associated with carotid artery plaque. A lumbar radiograph is more convenient than CT, has less radiation exposure, and is routinely performed in orthopedic examinations. Further longitudinal research is desirable to evaluate the impact of similar screening in healthy volunteers.
